# An Evaluation of the Responsiveness and Discriminant Validity of Shoulder Questionnaires among Patients Receiving Surgical Correction of Shoulder Instability

**DOI:** 10.1100/2012/410125

**Published:** 2012-05-01

**Authors:** Kyle A. R. Kemp, David M. Sheps, Lauren A. Beaupre, Fiona Styles-Tripp, Charlene Luciak-Corea, Robert Balyk

**Affiliations:** ^1^Orthopaedic Research, Alberta Health Services, Capital Health Region, 1F1.52 WMC, 8440-112 Street, Edmonton, AB, Canada T6G 2B7; ^2^Department of Surgery, University of Alberta, 1F1.52 WMC, 8440–112 Street, Edmonton, AB, Canada T6G 2B7; ^3^Department of Physical Therapy, University of Alberta, 3-71 Corbett Hall, Edmonton, AB, Canada T6G 2G4; ^4^Department of Rehabilitation Medicine, Covenant Health-Grey Nuns Hospital, 110 Youville Drive West, Room 1107, Edmonton, AB, Canada T6L 5X8

## Abstract

Health-related quality-of-life (HRQL) measures must detect clinically important changes over time and between different patient subgroups. Forty-three patients (32 M, 13 F; mean age  =  26.00  ±  8.19 years) undergoing arthroscopic Bankart repair completed three validated shoulder questionnaires (Western Ontario Shoulder Instability index (WOSI), American Shoulder and Elbow Surgeons Standardized Shoulder Assessment form (ASES), Constant score) preoperatively, and at 6, 12, and 24 months postoperatively. Responsiveness and discriminant validity was assessed between those with a satisfactory outcome and those with (1) a major recurrence of instability, (2) a single episode of subluxation, (3) any postoperative episode of instability. Eight (20%) patients reported recurrent instability. Compared to baseline, the WOSI detected improvement at the 6- (*P* < 0.001) and 12-month (*P* = 0.011) evaluations. The ASES showed improvement at 6 months (*P* = 0.003), while the Constant score did not report significant improvement until 12 months postoperatively (*P* = 0.001). Only the WOSI detected differential shoulder function related to shoulder instability. Those experiencing even a single episode of subluxation reported a 10% drop in their WOSI score, attaining the previously established minimal clinically important difference (MCID). Those experiencing a frank dislocation or multiple episodes of subluxation reported a 20% decline. The WOSI allows better discrimination of the severity of postoperative instability symptoms following arthroscopic Bankart repair.

## 1. Introduction

In orthopaedics and rehabilitation, the assessment of health-related quality of life (HRQL) is an important outcome to consider when assessing the effectiveness of various interventions [1, 2]. Validated patient-reported questionnaires are commonly used to obtain the patients' perception of the limitations that are associated with various musculoskeletal conditions. A number of joint and disease-specific HRQL measures now exist for many of the main conditions of the shoulder, including rotator cuff tears and recurrent instability [1, 3]. Some measures were developed using rigorous and accepted methodology [4, 5], while others were developed based on clinical validity and utility [4, 6, 7]. Many of these instruments have been assessed for their reliability and validity and to a lesser degree, their responsiveness, or ability to assess change over time and have been found to be adequate [8–10]. However, it has been hypothesized that the less rigorously developed questionnaires may not be as responsive or as discriminative, when compared with newer, condition-specific questionnaires [1, 4, 6, 11].

Questionnaire selection may play an important role in determining the extent of recovery or disability as well as detecting differential recovery among patients, particularly when only modest differences in outcome may be expected (e.g., comparison of different surgical interventions). Thus, it is important that appropriate HRQL assessment tools are chosen in order to detect clinically important changes (1) over repeated postoperative time periods (responsiveness) and/or (2) among different patient subgroups (discriminant validity).

Patients with chronic posttraumatic shoulder instability commonly experience significant impairment during work, sports, or while performing activities of daily living (ADL). Often times, their limitations are great enough to warrant surgical intervention [12, 13]. The short-and long-term success of these techniques has been widely demonstrated within the literature, with the incidence of postoperative recurrent dislocation being less than 10% and of recurrent postoperative instability (i.e., recurrent dislocation or subjective sense of subluxation being less than 20%) [13]. Given that this population experiences significant functional gains following surgery, we felt that this condition was an appropriate one to compare how selected shoulder questionnaires perform in (1) responsiveness and (2) discrimination among preselected subsets of patients.

Therefore, the goal of the present study was to perform a prospective evaluation of the performance of three different questionnaires commonly used to evaluate outcomes following surgical correction of chronic shoulder instability: (1) the disease-specific Western Ontario Shoulder Instability index (WOSI), (2) the shoulder-specific American Shoulder and Elbow Surgeons Standardized Shoulder Assessment form (ASES), and (3) the shoulder-specific Constant score. The primary aim was to compare the responsiveness of these three instruments over multiple postoperative time periods. The secondary aim was to compare the instruments' abilities to discriminate among three subsets of subjects: (1) those with major recurrence of instability (e.g., frank dislocation), (2) those with a single episode of subluxation, and (3) those with any recurrence of instability (i.e., all subjects who reported any of the above described symptoms) in the first 2 years relative to those subjects who did not report a recurrence of instability.

We hypothesized that the disease-specific questionnaire (WOSI) would be more responsive to change over time when compared to the two shoulder-specific instruments; the ASES and the Constant score. Further, we hypothesized that the WOSI would be better able to discriminate between those who had a successful outcome and those who experienced any recurrence of instability symptoms following surgical repair of their Bankart lesion compared with either of the two shoulder-specific questionnaires.

## 2. Materials and Methods

Between 2001 and 2007, a total of 43 subjects (32 men, 11 women; mean age = 26.0 ± 8.2 years) with unilateral, symptomatic, recurrent posttraumatic anterior shoulder instability were included in our prospective study. To be included in the study, subjects had to have symptoms of anterior glenohumeral instability that significantly affected their ability to function in daily life and a positive apprehension test. Subjects were excluded if they had undergone previous shoulder surgery, had multidirectional instability, or were unable to speak or read the English language.

Subjects underwent an arthroscopic Bankart repair using bioabsorbable Suretac anchors (Smith & Nephew Endoscopy, Andover, MA). The surgical procedures were performed by one of two subspecialty trained arthroscopic surgeons. To be eligible at surgery, subjects had to present with labral pathology indicative of a Bankart lesion (injury at the 3–6 o'clock position). Those with a superior labral anterioposterior (SLAP) lesion were also included. Those with only a SLAP lesion or those with a full or partial thickness rotator cuff tear were excluded.

Prior to surgery, baseline demographic information (age, sex, smoking status) and shoulder/injury characteristics (arm dominance, arm injured, level of sport competition played (when applicable), chief complaint relative to injury) were gathered.

All subjects completed a standardized rehabilitation protocol. Subjects were immobilized in a simple sling for the first 4 to 6 weeks. During this time, external rotation and abduction were not permitted; however, active and active-assisted forward flexion and internal rotation range of motion (ROM) exercises were encouraged. Following this initial period of immobilization, progressive ROM and strengthening exercises commenced. Subjects were permitted to return to full work and sports activities once they had full ROM, strength, and functional stability of the involved shoulder.

As part of the prospective study, subjects underwent a musculoskeletal examination, including ROM and strength testing, by a licensed physiotherapist preoperatively, and at 6, 12, and 24 months postoperatively. During these evaluations, subjects also completed three shoulder questionnaires: the WOSI, the ASES, and the Constant score, which are the focus of the present paper.

### 2.1. Instruments

The* WOSI* is a condition-specific questionnaire designed for use with patients who have shoulder instability [5]. It is comprised of 21 self-reported items, divided into 4 sections; physical symptoms (10 items), sport/recreation/work function (4 items), lifestyle function (4 items), and emotion function (3 items). Each item is scored on a 100-mm visual analog scale (VAS), with the best possible raw score being 0 points and the worst being 100 points. Therefore the best possible cumulative score is 0, indicating no disease, while the worst one is 2,100, indicating the presence of extreme disease (i.e., instability). Cumulative scores may be reported as well as subscale scores. In the present study, only cumulative scores are reported and were standardized to a 0–100 scale where 100 indicated no shoulder dysfunction related to instability. This scale has been shown to be valid, reliable, and responsive [4, 14]. Further, a minimally clinically important difference (MCID) of 10%, that is, the minimal difference in the WOSI score that has to occur for a patient to rate their shoulder as having changed, has been established [5].

The patient self-evaluation section of the ASES is a shoulder-specific instrument and is comprised of 11 items, which are divided into two areas; pain (1 item) and function (10 items) [7]. The pain item consists of a 10 cm visual analog scale (VAS), which asks the patient “how bad is your pain today?”. The 10 cm scale is divided into 1 cm increments and is anchored with verbal descriptors (“no pain at all”, “as bad as it can be”). The items comprising the function area of the ASES include 10 questions pertaining to activities of daily living. Patients are asked to indicate their ability to complete a given list of activities using a four-point Likert scale (0, unable to do; 1, very difficult; 2, somewhat difficult; 3, not difficult). These range from simple activities, such as putting on a coat, and combing hair, to more demanding ones, such as lifting ten pounds above shoulder level and throwing a ball in an overhand fashion. The final two items of the function section pertain to the patient's usual work and sports. For these items, patients are asked to select personal work and sports activities which are important to them, (i.e., ones in which they are likely to participate in and that they take part in frequently enough that they may provide a comment above that activity's relative difficulty throughout the year). To obtain the final score out of 100, the pain score (maximum of 10) is multiplied by five (for a total of 50), and the cumulative activity score (maximum of 30) is multiplied by 5/3 (for a total of 50), so that the pain and activity elements of the questionnaire are equally weighted. No published data exists to support this weighting scheme. Although there is limited information on development of the ASES, it has been shown to be valid, reliable and an MCID of 6.4 points has been established for this score [8, 9].

The Constant Score is the most widely used shoulder evaluation questionnaire in Europe [15], and is a shoulder-specific instrument. The score is a combination of an objective physical examination (65 points) and a subjective patient self-evaluation (35 points) [6]. The physical examination component includes a range of motion assessment (forward elevation, lateral elevation, internal rotation, and external rotation), worth a total of 40 points (maximum of 10 points for each motion). The remaining 25 points are attributed to the strength assessment, where patients are awarded one point for each pound of pull that the patient can resist in abduction. Therefore, the total possible score on the Constant Score is 100 points (best possible score = 100, worst possible score = 0). Although there is very limited data on the development of the instrument, the Constant score has been shown to be reliable, valid and responsive in assessing the impact of shoulder interventions [10]. No MCID has been established for this scale.

### 2.2. Outcomes

The primary outcome was the ability for the instruments to detect change in subjects' condition over multiple time periods (responsiveness). The secondary outcome was the ability for the instruments to detect differences in outcomes among three subgroups with recurrent instability relative to those who reported no recurrent instability (discriminant validity).

### 2.3. Definitions of Recurrence of Instability

We defined three subsets of subjects prior to starting the discriminant validity analysis. We were interested only in recurrent instability rather than any shoulder reinjury. The first subgroup was made up of those who had a major re-occurrence of instability, defined as a frank dislocation during sports or ADL (shoulder dislocation that required medical intervention to relocate) or multiple episodes of subluxation (did not require medical intervention to relocate, but each subluxation episode produced symptoms similar to preoperative symptoms of instability). The second subgroup of subjects was made up of those who experienced a single episode of subluxation (i.e., did not require medical intervention to relocate, but had one episode of subluxation that produced symptoms similar to preoperative symptoms of instability) while the final subgroup combined the initial two groups and looked at subjects who had any recurrence of instability (i.e., at least 1 episode of subluxation and/or a frank dislocation).

### 2.4. Analysis

Statistical analysis was performed using statistical packages for the social sciences (SPSS), version 18.0 (SPSS Inc., Chicago, IL). Descriptive statistics (mean, standard deviation, range) were calculated for all variables, in order to determine statistical significance, an alpha level of *α* < 0.05.

To examine responsiveness, a repeated measures analysis of variance (ANOVA) was undertaken over four time periods with a post-hoc contrast analysis of each interval for the three questionnaires. For the discriminant analysis, baseline comparisons were made between those who experienced a satisfactory outcome and those patients who experienced (1) a major recurrence of instability as previously defined, (2) a single episode of subluxation, and (3) any postoperative episode of instability to look for any systematic baseline differences among these patients. Then, scores at 6 months and at 2 years were compared between those without any adverse stability events and each of these three subgroups of patients as all reinjuries were reported after six-months postoperatively. An independent *t*-test analysis was performed on the change in each of the scores between the six-month and final evaluation to determine if the outcome measures were able to detect significant differences between each subgroup relative to those who reported a successful outcome at two years.

## 3. Results

### 3.1. Demographic and Injury Characteristics

Complete demographics are provided in Table 1. Subjects were predominantly male and right hand dominant. The average age of the participants was 26.0 ± 8.2 years. Many participants (*n* = 26; 60.5%) competed in competitive sports at the time of their injury. The indication for surgery was instability in 40 (93%) cases and instability with pain for the other three patients.

Forty (93%) patients were followed out to two years postoperatively. Three subjects reported a frank dislocation (*n* = 2) or multiple episodes of subluxation (*n* = 1). Five additional subjects reported a single episode of subluxation for a total of 8 (20%) subjects reporting recurring issues with instability.

### 3.2. Responsiveness

**Figure 1:**
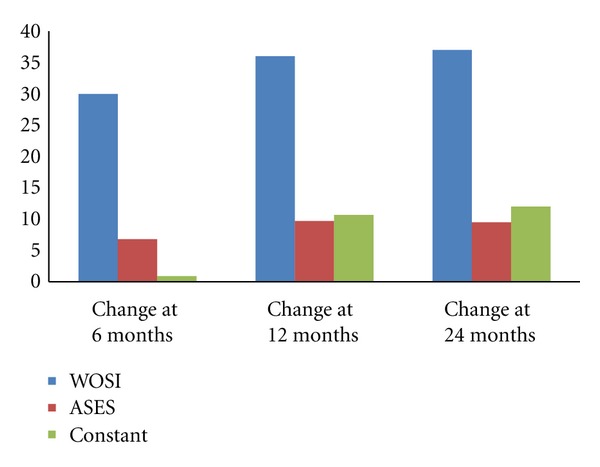
Mean change from preoperative evaluation over time of three common shoulder questionnaires.

Table 2 reports the scores for each of the outcome measures over time. The WOSI had a substantially lower preoperative score than either the Constant or ASES scores. The WOSI was also able to detect significant improvements in the subjects' shoulder symptoms at both the six-and 12-month evaluation, with no substantial changes noted in shoulder function between 12 and 24 months postoperatively (Table 2 and Figure 1). In contrast, the ASES showed a significant improvement between preoperative and six-month scores, but no significant improvements were detected after that time (Table 2 and Figure 1). The Constant did not report a significant improvement until 12 months postoperatively with no further improvements noted after the 12-month evaluation (Table 2 and Figure 1).

### 3.3. Discriminant Validity

The preoperative scores for subjects who had any recurrence of instability and those who were not significantly different for all three of the outcome measures (Table 3). However, the WOSI was able to detect significant differences in outcomes between subjects who had any reported episodes of instability with a score of reduction of at least 10 points reported between the pre- and post- reinjury assessment, including those subjects who reported only a single episode of subluxation. Those who reported a frank dislocation or multiple episodes of subluxation reported a mean reduction of over 20 points in the WOSI (Table 3). In contrast, the ASES was not able to detect changes in shoulder function in subjects who reported any kind of reinjury (Table 3). The Constant score appeared better able to detect group differences, but changes in the Constant score were less than nine points between pre- and post- reinjury (Table 3).

## 4. Discussion

In a cohort of 43 patients who underwent arthroscopic surgery for recurrent glenohumeral instability, three commonly used shoulder evaluation questionnaires were able to detect improvements in patients' postoperative shoulder function. However, as hypothesized, the WOSI was the most responsive of the three instruments and was able to detect incremental improvement over time. When compared to preoperative values, significant improvements in the WOSI were noted six months postoperatively with further substantial improvements between six and 12 months postoperatively. In contrast, the ASES scale noted significant functional improvements beginning at the six-month mark, but did not detect further improvements at either the 12- or 24-month assessment. The Constant score did not detect significant postoperative functional change until 12 months following surgery.

Further, in predefined subsets of patients who might be expected to report differential postoperative recovery, only the WOSI was able to discriminate differential shoulder function related to shoulder instability. Although only three (7.5%) patients were identified as a postoperative failure and five additional subjects reported recurrent symptoms of instability at the 24-month postoperative period, the WOSI score changed in alignment with the severity of the postoperative complication even with these limited numbers. A recurrent dislocation rate of approximately 10% has been reported in previous literature with rates of any instability recurrence being reported between 10 and 20%. These differences reported in the literature may reflect both the different definitions of recurrent instability that were used among studies as well as the difference in surgical techniques or rehabilitation programs.

We had over recurrent instability rates that were at the higher end of the reported range, which may reflect the poorer performance of the SureTac, a device that has mostly been abandoned in current practice, or may reflect the stringent measurement of recurrent instability symptoms. Subjects who we considered to have a major recurrence (frank dislocation or multiple episodes of subluxation) reported a 20% decline in their WOSI score, well beyond the established MCID [5]. The five additional subjects who reported only a single episode of subluxation in the initial 24 months postoperatively reported a more modest, but still detectible reduction in their function as measured by the WOSI that attained the previously established MCID [5]. In contrast, neither the ASES nor the Constant were able to discriminate in outcomes among these subgroups.

These findings were not unexpected, as the items on the WOSI focus on restrictions that are commonly reported by subjects experiencing shoulder instability. Our findings expand upon previous research, which showed that the WOSI was more responsive at the 2-week and 3-month postoperative periods, when compared with several other measures of shoulder function, including the ASES and Constant Score [5, 14], as well as measures of general health such as the 12-Item Short-Form Health Survey [14]. To our knowledge, however, this is the first time that the ability of questionnaires to discriminate among predefined subsets of patients has been examined. It was surprising to see the level of discrimination attained by the WOSI, where those who had only a single episode of recurrent instability had a detectible change in function. This level of discrimination would be very useful in studies that do direct comparisons of different surgical techniques for shoulder instability when only modest outcome differences are expected.

Previous literature has hypothesized that the ASES may have poor responsiveness, especially among patients with better function [16–19]. As each item is scored based on difficulty associated with certain tasks, it may be relatively easy to improve one's ASES score by one point, creating a potential ceiling effect within certain patients [16, 18]. Conversely, the Constant score has been reported to have substantial floor effects because subjects may have difficulty completing the strength testing due to the prescribed testing position [19]. However, in a previous study, Conboy et al. reported that all subjects in a study of 25 patients with recurrent instability scored well on the Constant score even prior to intervention [17]. Our results replicate this finding in that preoperatively, the average Constant score did not indicate a great deal of shoulder dysfunction, leaving very little room for postoperative improvement.

The study has a number of strengths including the high rate of followup out to 24 months and a prospective study design with preoperative assessment that diminishes the amount of bias that may occur in nonrandomized studies. Unlike most previously published series, the prospective data collection, with outcomes established a priori, has allowed assessment of subjective shoulder function at regular postoperative intervals. This methodology has allowed us to examine recovery over time to determine when the majority of recovery occurs and when shoulder function appears to stabilize over time. Further, it also allowed us to detect when subjects experience a loss of function due to recurrent symptoms or failure of the intervention.

However, there are some notable limitations to this paper. We did not have a comparison group, and only a small number of subjects experienced an adverse postoperative outcome. Because we had such a small study group, our results should be applied with some caution and these measurements should be repeated in larger studies examining the management of recurrent instability. Despite these limitations, our study demonstrates the importance of considering expected outcomes and choosing instruments that will allow the best discrimination amongst various patient subsets and that can monitor small change in shoulder function over time.

## 5. Conclusion

Our findings suggest that similar to other studies examining psychometric properties of common shoulder evaluations, all three of the instruments could be called responsive instruments. However, when compared with other items, the WOSI is the most appropriate subjective questionnaire for detecting postoperative functional change in recurrent shoulder instability population both over time and between groups and should be selected over other measures in this clinical population.

## Figures and Tables

**Table 1:** Demographic & preinjury characteristics of sample with shoulder instability.

Variable	All *n* = 43	No recurrence *n* = 32	Recurrence of instability *n* = 8
Demographics			
Mean age in years (SD)	26.0 (8.2)	25.5 (7.8)	28.4 (10.0)
Male sex (%)	32 (74)	24 (75)	6 (75)
Smokers (%)	8 (19)	5 (17)	2 (25)
Shoulder characteristics			
Right side injury (%)	25 (58)	22 (62)	3 (38)
Right hand dominance (%)	39 (91)	28 (88)	8 (100)
Competitive sport level			
International (%)	1 (2)	1 (3)	0 (0)
National (%)	1 (2)	1 (3)	0 (0)
Provincial (%)	7 (16)	5 (17)	1 (12)
Local (%)	17 (40)	11 (34)	4 (50)
Not applicable (%)	17 (40)	14 (43)	3 (38)

**Table 2:** Responsiveness (change over time) of three common shoulder questionnaires.

WOSI	Preoperative	6-month postoperative	12-month postoperative	24-month postoperative
Score (mean (standard deviation))	48.1 (19.8)	78.1 (16.6)	84.1 (14.6)	85.1 (14.6)
*P* value*	—	<0.001	0.011	0.69

ASES	Preoperative	6-Month Postoperative	12-Month Postoperative	24-month postoperative

Score (mean (standard deviation))	84.7 (7.8)	91.5 (10.3)	94.4 (4.9)	94.2 (7.7)
*P* value*	—	0.003	0.10	0.88

Constant	Preoperative	6-Month Postoperative	12-Month Postoperative	24-month postoperative

Score 9(mean (standard deviation))	81.6 (15.9)	82.5 (14.2)	92.2 (7.7)	93.6 (6.6)
*P* value*	—	0.86	0.001	0.41

*Using Post Hoc contrasts (repeated) to measure changes between intervals.

**Table 3:** Discriminant validity of three common shoulder questionnaires.

WOSI	Preoperative*	*P* value	6-month postoperative*	24-month postoperative*	Difference*	*P* value
Recurrence						
Yes	47.3 (17.2)	0.96	71.4 (27.1)	50.8 (6.0)	−20.6 (21.0)	0.02
No	46.8 (17.2)		75.3 (17.7)	84.4 (17.0)	9.2 (16.6)	
Single subluxation						
Yes	79.8 (9.3)	0.73	76.3 (25.4)	65.7 (27.1)	−10.5 (9.1)	0.02
No	80.1 (13.6)		74.8 (16.8)	85.4 (15.2)	10.6 (17.4)	
Any Reinjury						
Yes	44.6 (20.8)	0.86	74.9 (23.6)	61.5 (23.4)	−13.4 (12.4)	< 0.001
No	45.9 (17.6)		75.1 (16.6)	88.0 (12.1)	13.0 (15.0)	

ASES	Preoperative*	*P* value	6-month postoperative*	24-month postoperative*	Difference*	*P* value

Recurrence						
Yes	84.5 (11.5)	0.75	88.0 (5.2)	88.2 (5.2)	0.2 (00)	0.63
No	82.7 (9.0)		88.2 (15.7)	92.5 (14.5)	4.3 (11.7)	
Single subluxation						
Yes	79.8 (9.3)	0.96	82.1 (30.7)	79.5 (32.0)	−2.6 (1.8)	0.17
No	80.1 (13.6)		89.2 (11.6)	94.4 (7.7)	5.2 (12.3)	
Any reinjury						
Yes	81.6 (9.6)	0.73	83.8 (25.3)	82.0 (25.6)	−1.8 (2.0)	0.14
No	79.8 (13.8)		89.2 (12.0)	94.8 (7.7)	5.6 (12.7)	

Constant	Preoperative*	*P* value	6-month postoperative*	24-month postoperative*	Difference*	*P* value

Recurrence						
Yes	80.1 (18.7)	0.98	91.2 (0.28)	83.8 (18.0)	−7.5 (17.7)	0.08
No	79.9 (12.8)		82.7 (17.2)	92.1 (13.4)	9.4 (12.4)	
Single subluxation						
Yes	71.9 (17.8)	0.41	74.8 (35.3)	75.7 (31.8)	1.9 (3.6)	0.31
No	78.3 (16.0)		84.8 (12.6)	94.0 (6.6)	9.2 (13.8)	
Any reinjury						
Yes	75.0 (17.3)	0.63	79.6 (28.8)	78.4 (26.3)	−1.2 (9.7)	0.05
No	78.1 (16.0)		84.2 (13.0)	94.8 (4.8)	10.6 (12.9)	
